# Frequency spectrum recurrence analysis

**DOI:** 10.1038/s41598-020-77903-4

**Published:** 2020-12-04

**Authors:** Guênia Ladeira, Norbert Marwan, João-Batista Destro-Filho, Camila Davi Ramos, Gabriela Lima

**Affiliations:** 1 Faculty of Mechanical Engineering, Federal University of Uberlândia, Uberlândia, Minas Gerais Brazil; 2 Potsdam Institute for Climate Impact Research, P.O. Box 601203, 14412 Potsdam, Germany; 3 Interdisciplinary Centre for Dynamics of Complex Systems, University of Potsdam, 14415 Potsdam, Germany; 4 Faculty of Electrical Engineering, Federal University of Uberlândia, Uberlândia, Minas Gerais Brazil

**Keywords:** Computational models, Computational neuroscience, Data acquisition, Data processing, Biomedical engineering, Electrical and electronic engineering, Computational neuroscience, Neural circuits, Visual system, Brain injuries

## Abstract

In this paper, we present the new frequency spectrum recurrence analysis technique by means of electro-encephalon signals (EES) analyses. The technique is suitable for time series analysis with noise and disturbances. EES were collected, and alpha waves of the occipital region were analysed by comparing the signals from participants in two states, eyes open and eyes closed. Firstly, EES were characterized and analysed by means of techniques already known to compare with the results of the innovative technique that we present here. We verified that, standard recurrence quantification analysis by means of EES time series cannot statistically distinguish the two states. However, the new frequency spectrum recurrence quantification exhibit quantitatively whether the participants have their eyes open or closed. In sequence, new quantifiers are created for analysing the recurrence concentration on frequency bands. These analyses show that EES with similar frequency spectrum have different recurrence levels revealing different behaviours of the nervous system. The technique can be used to deepen the study on depression, stress, concentration level and other neurological issues and also can be used in any complex system.

## Introduction

The sensory organs of the nervous system capture external information, the brain then processes this information and generates stimuli in the body. Knowledge of brain functions aids in improving control of activities body, as well as the treatment of disease.

The first collection and observation of electrical signals from the human encephalon was performed by Hans Berger^1^. These signals were later classified into frequency bands resulting of different neurological states^2^. Assessing the relationship between brain function and EES the functional connectivity of the brain was verified between functional magnetic resonance imaging (fMRI) and electroencephalogram EEG^3^.

The nervous system has been analysed under different aspects. With the focus being on health we have jobs like, the amplitude of the alpha wave can be used as a biological marker for identifying states of depression^4^. Upon analysing type alpha-1 (8 to 10 Hz) and type alpha-2 (10 to 12 Hz), in experiments with the eyes closed and open, it was found that individuals suffering from depression have lower amplitude waves than those without depression^4^.

Preliminary results confirm that the analysis of signals from a single EEG channel using a combination of measures can identify the level of depression^5^. The quality of life during cognitive aging was analysed by means of reactivity to eyes opening^6^.

Many studies using the alpha waves from the EEG, as their amplitude are highlighted in one or more frequency bands, as well as the fact that the magnitude from these oscillations varies under different circumstances^7^. Alpha waves are extremely coherent over short intervals of time; however, the Lyapunov exponents demonstrate that the variability of the brain increases sharply over long periods of activity^8^, which justifies the analysis by the frequency spectrum over long time series.

Through observation of the functional part of the brain a number of different studies have been developed. Biofeedback training, used to alter the heart rate, resulted in changes to the EEG with increases in alpha waves and decreases in the theta waves mainly in the right hemisphere, the prefrontal region^9^.

Research by Klimesch analysed the evidence for alpha and theta waves acting upon the reasoning of memory^10^. In a decompression chamber, the EEG was applied to the investigation of plasticity in the nervous system in the absence of weight, by means of visuo-attentional conditions imposed upon the volunteer before a visuomotor task^11^. The increase in power from the alpha wave associated with sleep deprivation decreased the ability to respond to stimuli^12^. A study was performed on the involvement of the alpha band in maintaining auditory working memory (AWM)^13^.

The electrical signals in the nervous system reveal that it holds a nonlinear and variable behaviour in time, as presented in some studies that go on to reveal such features. Nonlinear approaches are more adequate for measuring intrinsic dynamics during sleep, by means of EEG^14^. The filtered alpha rhythm does not have the same dynamics as white noise filtered on the same spectrum band. And, the moment that daily learning influences the connections between neurons the brain can be considered as a variable system, over the period that memories can be altered^15^. The neurological system possesses long-range temporal correlations (LRTC) in fluctuations of thousands of cycles. During rest, high levels of LRTC on the alpha band in the sensorimotor region predict a well-executed attention activity^16^.

It is acknowledged that alpha waves of the occipital lobe possess greater amplitude in the waking state with the eyes closed than when open, which results from the sensory perception of the eyes, as well as the propagation of information through the nervous system to the memory in the cerebral cortex.

Methods used for analysing the recurrence of behaviour in dynamic systems have been improved over recent years. As for example, an emotion recognition system based on the geometric analysis of the autonomous nervous system was generated using lagged Poincaré plots^17^.

The recurrence quantification analysis (RQA) offers the quantitative analysis of recurrence properties of dynamical systems in time and is widely used for biological systems. In a previous study, we have evaluated the level of quantifiers of cross-recurrence between frequency and amplitude variation of the electrical stimulus of the heart with respiratory control^18^. The EEG recurrence percentage markers reliably quantified two aspects related to sleep quality, sleep depth and sleep fragmentation^8^. A scoring system of the spectral sleep component was investigated in a simple and objective test^19^. The study examined heart rate synchronization during psychological counselling, demonstrating the applicability of recurrence analysis to complex data^20^. Recurrences can also be used to study the coupling mechanisms in physiological systems; such in the cardiorespiratory system^21^.

Recurrence is usually considered as repeating states in the time domain. However, recurrences can also appear in domains different than time. For example, several approaches of spatial recurrence analysis have been suggested^22–25^ and applied to physiological, ecological, and engineering data. Recurrence patterns are also apparent in the frequency domain, but have not yet been considered so far.

In this work, we will present the Frequency Spectrum Recurrence Analysis technique using electro-encephalon signals (EES) collected in the occipital region in two states, one with open eyes and the other with closed eyes from a group of seven people.

The aim is to characterize the alpha band filtered EES of the two states using standard recurrence analysis and frequency recurrence analysis and compare both techniques. Moreover, we created the quantifiers Recurrence Concentrations on Frequency ${Rc}_{f}$ and Recurrence Concentrations on Frequencies Bands ${Rc}_{(i)}$ generating new ways of analysing recurrence in frequency.

First, we analyse the signals by statistical analysis, i.e., mean amplitude and standard deviation, inter-states correlations, and by calculating the largest Lyapunov exponents. By this study, we demonstrate the new idea of analysing signals by recurrence features in the frequency domain and evaluate this method by comparing with standard data analysis techniques.

## Methods

### Data collection

Electro-encephalon signals (EES) were collected by Camila Davi Ramos^26^. Our study was present to participants and informed consent of participation were signed by them. All participants were adults aged over 18 years. All experimental protocols were approved by the Research Ethics Committee of the Federal University of Uberlândia, which officially accepted the development of our research (Protocol No. 54781615.6.0000.5152). All collection methods were carried out in accordance with relevant guidelines and regulations.

Participants were selected using the criteria: (a) no illness at the time of data collection and (b) not ingested substances that could affect the nervous system, such as coffee, tea, alcohol or drugs, within the last 48 h prior to testing.

Data collection was performed at the Neurology Department of the Clinical Hospital of the Federal University of Uberlândia using BrainNet BNT-EEG equipment. Data for each state were collected for 180 s. The electroencephalogram protocol used was 10/20. A neurologist validated the tests and checked the data quality.

In this work, EES samples from the cortex occipital region of seven participants will be analysed. The EES data collection was carried out with each participant in the two states (a) open eyes and (b) closed eyes in alert state. The electrodes used are identified by O1, Oz and O2.

### Pre-processing

The EES collected were prepared for numerical analyses. The signals from the open eyes and closed eyes were separated in epochs of 120 s each. These epochs were organised in different files and identified by participant code, state and electrode.

The signals were digitally filtered using a band pass filter from 7.5 to 12.5 Hz of the type finite impulse response and with order 1780 filter. The signals after filtering showed no change in amplitude in the passage band but were attenuated by – 60 to – 70 dB outside the passage band.

### Signal analysis with known techniques

A first analysis of the data was conducted using basic descriptive statistics, such as mean and standard deviation.

Pearson correlations were also calculated. This provides the level of association or similarity of behaviours between two signals by means of their covariance and standard deviations. The correlations were calculated between the signals with open and closed eyes. The correlations between the electrodes at the occipital regions O1, Oz and O2 were also verified.

After characterising the signals using linear techniques, we used nonlinear techniques. The largest Lypunov exponent λ was calculated according to Parlitz^27^ to compare the variability of the time-series signals in the reconstructed phase space. The parameters used for this analysis included the time delay τ and the embedding dimension D. According to Fraser and Swinney^28^, the optimal delay τ corresponds to the first local minimum of the mutual information. Embedding dimensionn D was selected by using the Cao method^29^. λ was then calculated for all participants and for all states using the same parameters of τ = 6 and D = 4.

Next, recurrence quantification analysis RQA was carried out. Basic measurements based on the diagonal lines of the recurrence matrix, namely, DET, L and ENT, were calculated. The determinism quantifier (DET) is the fraction of points in the recurrence matrix that form diagonal lines. DET can be interpreted as the predictability of a system and is, for example, higher for periodic dynamics than for chaotic processes; for stochastic dynamics it has low values.

The diagonal lines lengths quantifier (*L)* is the mean length of the diagonal lines in the recurrence matrix and corresponds to the average time of stability of the system.

The Shannon entropy^30^ (ENT) of the diagonal line lengths distribution reflects the complexity of the line length structure of the recurrence matrix. The value of ENT is small for noise, indicating their low complexity^31^. Persistence or chaotic dynamics increases the variation of line lengths in the recurrence plot (RP), thus, increasing the ENT values. A special case is periodic dynamics. Due to the boundary effect of the finite-size RP, diagonal lines of periodic dynamics have different length and the corresponding ENT values are higher than that for noise, requesting a border correction schema^32^. However, as we do not expect here periodic dynamics and continuous diagonal lines crossing the RP's border, such correction schema is not necessary in our application.

The adopted configuration for the recurrence analyses calculation were D = 4, τ = 6, a recurrence threshold ε that fixes the recurrence rate to 0.5, ${l}_{min}=$ 5, ${v}_{min}=$ 3, number of points *n* = 2000, sampling interval *dt* = 1/40 s, being RR recurrence rate, *n* number points, and *dt* time interval.

### Analysis of results

The confidence intervals (CI) of the mean of the results were calculated to generate ranges of values expressing the characteristics of a system state. The CI of 90% of the mean is given in Eq. (1). Because of the small sample size used in this work, analysis was carried out using Student's t-distribution, where $\stackrel{-}{X}$ is the sample mean, $\sigma$ is the standard deviation and $n$ is the size of the sample space. In the results section, the upper limit of CI is designated (up) and the lower limit (low). The CI depends on the sample size. For future work, we recommend performing a larger number of analyses to improve the CIs and state identification.

1 $$CI=\left(\stackrel{-}{X}-1.943*\frac{\sigma }{\sqrt{n-1}};\stackrel{-}{X}+1.943*\frac{\sigma }{\sqrt{n-1}}\right)$$

### Frequency spectrum recurrence analysis

The time series were transformed into the frequency domain by the Fast Fourier transform algorithm. Next, recurrence matrices were calculated from these frequency signals, according Eq. (2), where $\varepsilon$ is the recurrence threshold, $\Vert \cdot \Vert$ is the Euclidean norm, and $\theta$ is the Heaviside function:

2 $${R}_{\left({f}_{i},{f}_{j}\right)}= \theta \left(\varepsilon -\Vert {f}_{i}- {f}_{j}\Vert \right), i, j 1, \dots , n.$$

The recurrence matrix now is formed pairs of frequency values that are close in amplitude. In contrast to the regular recurrence matrix, where the axes represent time points, here the axes of the recurrence matrix represent frequency values. Two recurrence matrices were generated, one with threshold ε = 0.4, and the other with threshold ε = 0.95. The matrices points are subtracted, generating a new matrix with the points between thresholds. In this resulting matrix are the significant points of the signal, excluding noise with the elimination of points of very low amplitude, with ε = 0.4, and eliminating the very high amplitudes referring to the disturbance, with ε = 0.95.

The recurrence matrices were calculated using the following parameters: D = 3, τ = 1, fixes the recurrence rate, data length n = 902 and a sampling interval of df = 0.0056 Hz. D was selected because it provides a good resolution of the recurrence areas. τ was selected to encompass all points of the frequency’s series. The frequency spectra condense information of long time series, so can be considered to contain all details relevant to characterize a signal that are within the frequency thresholds defined for the analyses. The recurrence matrices have finally 900 × 900 points, and the recurrence interactions were performed at frequencies with intervals of df.

First, recurrence quantifiers relative to the diagonal lines DET, L and ENTR were calculated showing characteristics the recurrence matrices of the frequency series.

The recurrence matrices of the frequency signals present greater differences in recurrence on vertical/horizontal lines than on diagonal lines; thus, we developed a novel measure to analyse regions of recurrence in vertical direction.

### Recurrence concentrations on frequency ${Rc}_{f}$

The numbers of recurrence points were checked for each frequency in the matrix columns, being ${Np}_{f}$ number of points recurrence per frequency. The lengths of the sequences of recurrence points were verified for each frequency of the matrix, being ${{\mathrm{Ls}}_{\mathrm{f}}}_{\left(\mathrm{k},\mathrm{j}\right)}$ matrix with lengths of the series of recurrences in each frequency. The recurrence concentrations on frequency were verified for each recurrence frequency ${Rc}_{f}$ by calculating the lengths of the sequences ${Ls}_{f}$ divided by the number of sequences in each frequency ${Ns}_{f}$.

### Recurrence concentrations on frequencies bands ${Rc}_{\left(i\right)}$

We further introduced a measure that quantifies the recurrences by regions of the matrix. The quantifier Recurrence Concentrations on Frequencies Bands ${Rc}_{(i)}$ was created that calculates recurrences in a band, in this case of 0.25 Hz (corresponding to 45 sampling points) (Eq. 3).

3 $${Rc}_{(0.25)}=\sum_{j=[\mathrm{1,45}]}{{Rc}_{f}}_{(j)}$$

## Results

EES were analysed using several established techniques, and the results obtained were compared with those of the proposed technique.

### Statistical analysis of the time series

We compared statistically the signals that were collected whilst the eyes of the seven participants were closed and open. Alpha waves present greater amplitudes when the participants closed their eyes, with dimensionless mean of 38.963, whilst open eyes show a dimensionless mean of 26.628. The standard deviation calculated for closed eyes (10.317) is also higher than that calculated for open eyes (4.868).

### Correlation analysis of closed and open eyes

We analysed the correlations of the signals acquired by the O1 electrode between closed eyes and open eyes. The results show no substantial linear correlation. The highest correlation is 0.049 (participant P04), and the lowest is 0.002 (participant P01). The overall mean is 0.029. The correlations between electrodes O1, Oz and O2 located in the occipital region were high for all the participants, with a mean of 0.8.

### Analysis of the largest Lyapunov exponent

The variability of the time-series signals were quantified by the largest Lyapunov exponent (λ). The values with closed eyes are slightly higher owing to greater variability in the time-series signals. The results gathered from the statistical analyses are found on Table 1. The significance index in the comparison between the two states, demonstrates the difference in variability, ANOVA test-p (α = 0.1) single-factor resulted λ (p = 0.0071).

**Table 1:** Largest Lyapunov Exponent λ of occipital region electro-encephalon signals.

Variability of the time-series **λ**				
Group	Score	Sum	Mean	Variance
Closed eyes	7	29.162	4.166	0.0203
Open eyes	7	27.292	3.899	0.0274

### Recurrence quantification analysis of the EES time series

The EES have different characteristics in the two states. We calculated the recurrence quantifiers based on diagonal lines, including determinism (DET), mean length of diagonal lines (L), and entropy (ENTR). The lower limit (low) and upper limit (up) values of the mean 90% confidence intervals (CI) calculated from the EES of the seven participants are shown in Table 2. The CI ranges for the two events are different but partly overlap. ANOVA test-p (α = 0.1) single-factor resulted DET (p = 0.0962); L (p = 0.1332); and ENTR (p = 0.7099).

**Table 2:** Mean 90% confidence interval of the recurrence quantifiers calculated for the EES for closed and open eyes. (up) upper limit CI, (low) lower limit CI. (DET) determinism, (L) mean length of the diagonal lines in the recurrence matrix, (ENTR) entropy.

	CI mean—time EES			
	Closed eyes		Open eyes	
	Low	Up	Low	Up
DET	0.54	0.63	0.51	0.56
L	18.12	24.21	15.39	20.41
ENTR	2.63	6.34	2.59	5.46

### Frequency spectrum recurrence

**Figure 1:**
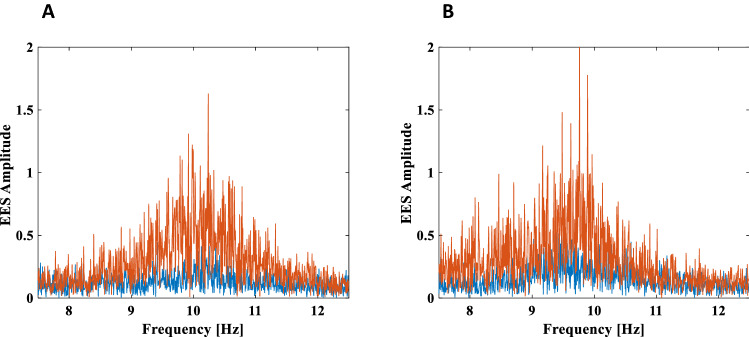
Frequency spectra of electro-encephalon signals of occipital region, **(A)** participant P01 and **(B)** participant P02; red corresponds to closed eyes, blue to open eyes.

After knowing characteristics of the signals analysed, we can observe on the frequency spectra the electro-encephalon signals of the participant P01 (Fig. 1A) and P02 (Fig. 1B). The red signals are from the participant with eyes open and the blue signals from the participants with eyes closed. The signals have higher power for closed eyes than for open eyes.

**Figure 2:**
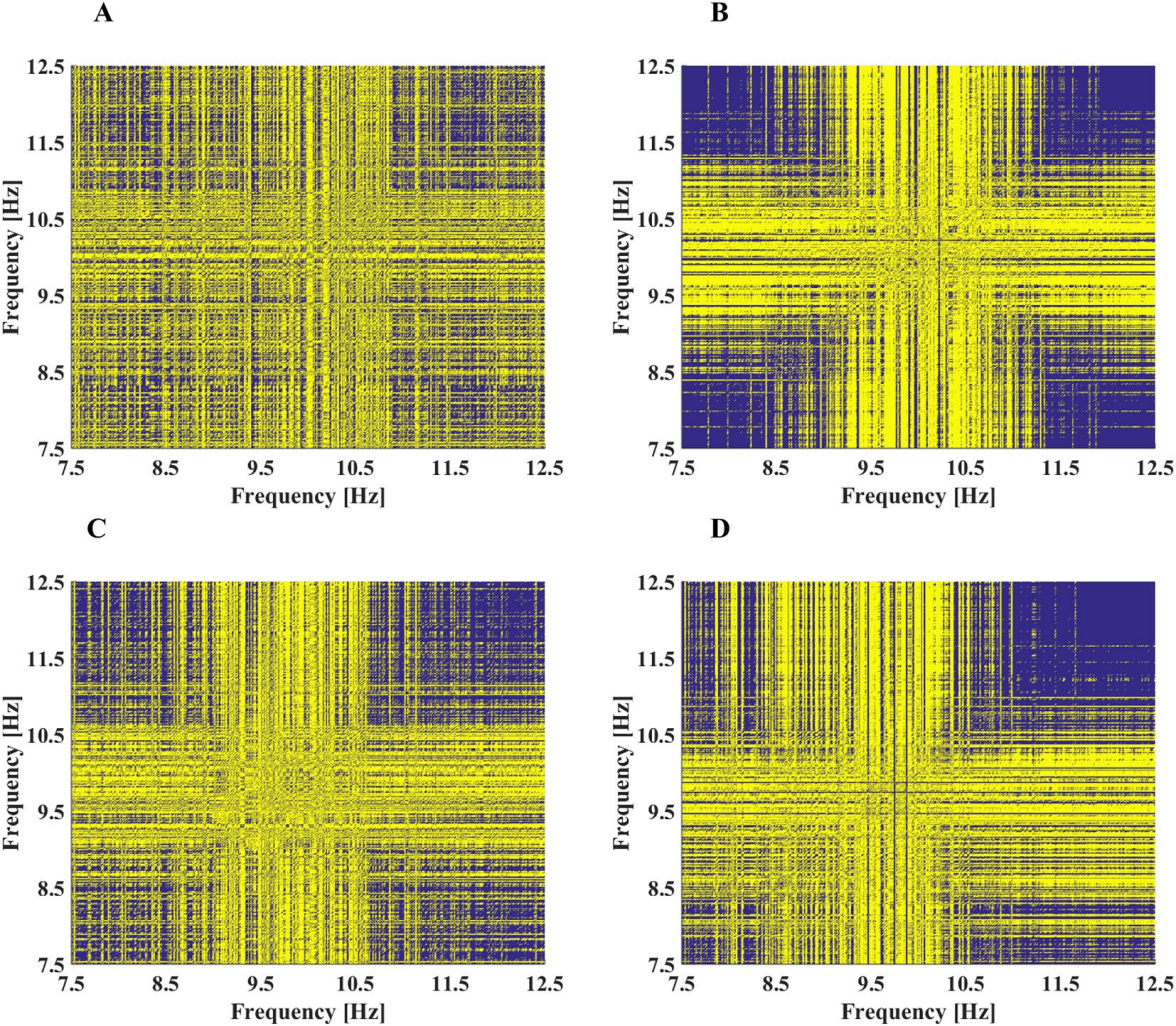
Recurrence matrices of the electro-encephalon signals from the Oz electrode of participants P01 and P02; (**A**) participant P01 with eyes open and (**B**) eyes closed; (**C**) participant P02 with eyes open and (**D**) eyes closed.

The next step is to calculate the recurrence matrices of the frequency spectra of the EES (Fig. 2). In general, we find that the recurrence matrix consists of more dispersed recurrence points (the yellow dots in Fig. 2) for open eyes (Fig. 2A,C) than for closed eyes (Fig. 2B,D). For closed eyes, the recurrence points aremore concentrated in the center of the plots. We also find differences in the appearance of recurrence plots between different participants (e.g., Fig. 2A,C), but the tendency to have more dispersed recurrence points for the state of open eyes than for closed eyes remains valid.

The recurrence matrices of the EES frequency spectra were further analysed using the recurrence quantifiers. For the results of the DET, L and ENTR we calculated the mean and standard deviations for the seven participants in both states (closed and open eyes), and finally the confidence intervals (CI) for the mean of these measures (Table 3). We find that all measures are higher for closed eyes than for open eyes. The confidence intervals of the mean for two groups do not overlap, indicating a significant difference of these measures for the two states. This was confirmed by an ANOVA single-factor test (with α = 0.1) with p-values for DET as p = 0.0079; for L as p = 0.0168; and for ENTR as p = 0.0131.

**Table 3:** Confidence interval (CI) of the mean of the recurrence quantifiers calculated from the EES spectra frequency for closed and open eyes; (up) upper limit CI, (low) lower limit CI. (DET) determinism, (L) mean length of the diagonal lines in the recurrence matrix, (ENTR) entropy.

	CI mean—frequency EES			
	Closed eyes		Open eyes	
	Low	Up	Low	Up
DET	0.67	0.78	0.58	0.65
L	5.02	6.62	4.28	4.91
ENTR	0.61	0.88	0.46	0.59

Next, we analysed the frequency spectra. As examples, we used the signals of P01 and P02 and also the average of the entire group of seven people. The mean amplitudes of the frequencies spectra in the band between 7.5 to 12.5 Hz of the three examples P01, P02 and the entire group are close with larger values for closed eyes than for open eyes, as we can see in line 1 of Table 4. Moreover, for the entire group the standard deviation of the mentioned frequency band is 0.05 for open eyes and 0.08 for closed eyes.

**Table 4:** Mean and standard deviation of the recurrence points concentration of the participants P01 and P02 and the group of seven people. The signals were collected from participants in two states, i.e., eyes open and eyes closed.

Recurrence concentrations on frequency—${Rc}_{f}$	P01		P02		Group mean	
	Open	Closed	Open	Closed	Open	Closed
1—mean of the signals on the frequency	0.12	0.28	0.15	0.30	0.17	0.35
2—mean of the recurrence points concentration ${Rc}_{f}$	2.92	5.88	3.83	4.75	3.45	6.40
3—standard deviation of the recurrence points concentration ${Rc}_{f}$	1.87	6.71	3.40	4.54	2.55	7.81

Looking at the similarities of the results, we deepened the analysis with the Recurrence Concentrations on Frequency, ${Rc}_{f}.$ We calculated the mean, line 2 of Table 4 and the standard deviation, line 3 of Table 4 of the ${Rc}_{f}.$

For participant P01, the mean frequency amplitude is 133.3% larger for closed eyes than for open eyes; mean ${Rc}_{f}$ is 101.4% larger and, the standard deviation of ${Rc}_{f}$ is 258.8% larger for closed eyes than for open eyes. We find for participant P02 mean frequency amplitude 100.0% larger for closed eyes than for open eyes; mean ${Rc}_{f}$ 24.0% larger and standard deviation of ${Rc}_{f}$ 33.5% larger for closed eyes.

The mean ${Rc}_{f}$ of all participants is 85.5% larger and the standard deviation is 206.3% larger for closed eyes (Table 4). Whereas the mean amplitude of the frequency spectra has close values, the recurrence concentrations ${Rc}_{f}$ vary widely between the participants.

**Figure 3:**
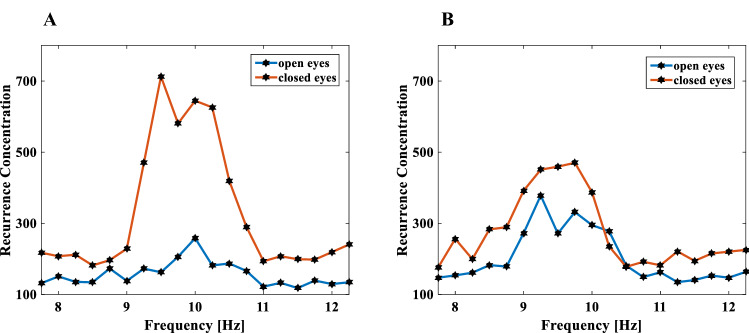
Recurrence concentrations on frequencies bands ${Rc}_{(0.25)}$ of electro-encephalon signals of the participants with eyes open and closed, (**A**) participant P01 and (**B**) participant P02. The points of the graphs are from bands of 0.25 Hz.

Finally, the Recurrence Concentrations on Frequency ${Rc}_{f}$, are grouped with the sum in 0.25 Hz bands, which is called Recurrence Concentrations on Frequencies Bands,${Rc}_{(0.25)}$. ${Rc}_{(0.25)}$ for participant P01 reveals a high recurrence concentration in the frequency band between 9 and 11 Hz (Fig. 3A), whereas participant P02 has a high recurrence concentration in a slightly lower frequency band, i.e., between 8.5 and 10.5 Hz (Fig. 3B).

The amplitude ${Rc}_{(0.25)}$ with open eyes for participant P01 peaks at 10 Hz and measures 258 (blue line—Fig. 3A). By comparison, the amplitude of ${Rc}_{(0.25)}$ with open eyes for participant P02 peaks at 9.25 Hz and measures 378.1 (blue line—Fig. 3B); hence, the frequency bands with the largest recurrence concentrations are different for these two participants. With the eyes closed, the difference of ${Rc}_{(0.25)}$ between P01 and P02 is greater, as can be seen in the graphs of Fig. 3 in the red lines.

Table 5 shows the percentage of increase in the recurrence concentrations of the alpha wave signals of the seven participants from eyes open to eyes closed. The results show a general increase in recurrence concentration for all participants but with different percentages.

**Table 5:** Percentage of increase in the concentration of recurrence on alpha waves when the participant changes eyes open to eyes closed.

	P01	P02	P03	P04	P05	P06	P07
Percentage of increase of recurrence concentration on the frequency	100.94	23.88	7.02	98.04	62.83	180.13	108.28

## Discussion

In this study, the authors herein present the novel Frequency Spectrum Recurrence Analysis by illustrating its application and potential for investigating neuronal effects in the nervous system. Here, the electro-encephalon signals (EES) from the occipital region of the brain of seven participants were analysed with their eyes open and closed.

The samples collected from the seven participants showed that the alpha waves of the occipital region commonly feature greater amplitude and standard deviation when the eyes are closed than eyes opened. However, the proportion of the amplitude by the standard deviation is lower for closed eyes (3.794) than for opened eyes (5.602), showing that closed eyes generate more organised signals and less variation.

The linear correlation between open and closed eyes was extremely low, confirming independence of the states. The correlation index indicates a relevant change in the nervous system with alteration of the alpha wave characteristics. The electric stimulus spreads throughout the occipital region, leading to a potential overlap of the EES signals with slight phase difference at these electrodes. This would be reflected by differences in the correlation for more distant electrodes. The frequency spectra of EES have high power in a certain band and low power with scattering in the remaining bands, which are characteristic of nonlinearities in the system.

To expand the analysis, the largest Lyapunov exponent was calculated for all participants. The embedding dimensions for the EES were calculated to be between four and eight, demonstrating the high variability of their orbits in the phase space^29^. EES from closed eyes presented larger amplitude cycles, which resulted in a shorter time delay (τ = 2) compared to open eyes (τ = 9). The largest Lyapunov exponent is higher for signals with eyes closed, thus possessing greater amplitude and greater variability of cycles when compared to those with eyes open.

Recurrence Quantification Analysis of the EES Time Series were performed for all participants in both states, resulting in only slightly larger values of the recurrence quantifiers for closed eyes. Greater entropy^30^ could be interpreted as a larger complexity in the operation of the occipital region of the brain, whereas longer diagonal line sizes might indicate a more stable behaviour. In this analysis, the confidence intervals of these measures overlap for the two states, and as such do not obtain a clear separation of the states.

The authors notice that the EES in the frequency have very approximate mean and standard deviations in each state. Furthermore, visually speaking, the frequency spectra are very similar. Thus, simple analyses on frequency do not show substantial differences between the EES.

The novel Frequency Spectrum Recurrence Analysis enables the acquired signals taken over long periods to be analysed in a single step, within the frequency spectrum.

This technical analysis identifies predominant patterns in amplitude and frequency variability. Each point in the recurrence matrix refers to a frequency within the range of 7.5 Hz to 12.5 Hz with a resolution of 0.006 Hz. The recurrence analyses were performed between threshold 0.4 that remove small amplitude recurrences considered as noise; and threshold 0.95 that remove signals high amplitude, related of disturbances that contaminated the data acquisition.

The diagonal lines of the recurrence matrices are formed by the correlation between the frequencies with amplitudes inside these thresholds. These show greater dispersion on the signals with eyes open than eyes closed. The level and the region of concentration reoccurrence are different for the individuals analysed.

Recurrence analyses are performed through quantifiers. The determinism quantifier (DET) is the fraction between the sequences of recurrence points greater than ${l}_{min}$ and sequences of any size. It shows how a frequency correlates with other frequencies within the thresholds. The DET quantifier is interpreted as the predictability index of the signal frequency composition.

The diagonal lines lengths quantifier *L*, i.e., the mean length of the recurrence sequences inside the thresholds provides information on the frequency amplitude stability when small frequency variations occur on the signal. The entropy (ENTR)^30^ of the recurrence on frequency is higher for more regular structures.

On Frequency Spectrum Recurrence Analysis the confidence interval of the mean (CI) of the quantifiers results does not result in overlap and create two range of different for each state. The ANOVA test-p demonstrated greater possibility of an equal mean for the recurrence results of the in time series than in frequency band.

New forms of recurrence analysis were created by quantifying the content of the vertical lines of the recurrence matrices in the frequency. The analysis of the recurrence concentration generates information concerning which part of the frequency band has more or less recurrence. The amplitude of recurrence can also be checked for each frequency or for a set of frequencies. The mean of the recurrence points concentration ${Rc}_{f}$ is different for each participant.

The quantifier Recurrence Concentrations on Frequencies Bands ${Rc}_{(0.25)}$ analyse the recurrence concentration on small frequency band intervals. These reduce the effect on the results of specific frequencies that had been removed from the analyses, due to contamination with disturbances. The results of ${Rc}_{(0.25)}$ showed differences in the level of concentration amplitude and the frequency range for the EES of the analysed participants.

The novel frequency spectrum recurrence analysis can create a breakthrough in research with use of the EES to detect depression^4^; or cognitive aging^6^; or even access to memory^10,13^, among other diverse experiments already being undertaken. Details of the characteristics of the functioning of the brain can be revealed through the analysis of the EES with its already known quantifiers DET, L and ENTR, and also with the new quantifiers created ${Rc}_{f}$ and ${Rc}_{(0.25)}$.

## Data Availability

The experimental data collected will be made available by request to the corresponding author by email.
